# Mo@GAA-Fe_3_O_4_ MNPs: a highly efficient and environmentally friendly heterogeneous magnetic nanocatalyst for the synthesis of polyhydroquinoline derivatives

**DOI:** 10.1039/d1ra00396h

**Published:** 2021-03-11

**Authors:** Mehraneh Aghaei-Hashjin, Asieh Yahyazadeh, Esmayeel Abbaspour-Gilandeh

**Affiliations:** Chemistry Department, University of Guilan Rasht 41335-1914 Iran; Young Researchers and Elites Club, Ardabil Branch, Islamic Azad University Ardabil Iran abbaspour1365@yahoo.com

## Abstract

Polyhydroquinolines were efficiently obtained from a sequential four-component reaction between dimedone or 1,3-cyclohexandione, ethyl acetoacetate, or methyl acetoacetate as a β-ketoester, aldehydes, and ammonium acetate, under the catalysis of Mo@GAA-Fe_3_O_4_ MNPs as a green, effective, recyclable, and environmentally friendly nanocatalyst. Due to its magnetic nature the prepared catalyst can be easily separated from the reaction mixture by an external magnet and reused several times without significant changes in catalytic activity and reaction efficiency. The catalyst was characterized using energy dispersive X-ray spectroscopy (EDX), X-ray diffraction (XRD), thermogravimetric analysis (TGA), Fourier transform infrared spectroscopy (FTIR), vibrating sample magnetometry (VSM), scanning electron microscopy (SEM), and transmission electron microscopy (TEM).

## Abstract 2

Polyhydroquinolines were obtained from a sequential four-component reaction between dimedone or 1,3-cyclohexandione, ethyl acetoacetate, or methyl acetoacetate as a β-ketoester, aldehydes, and ammonium acetate, with Mo@GAA-Fe3O_4_ MNPs as a green nanocatalyst.

## Graphical abstract

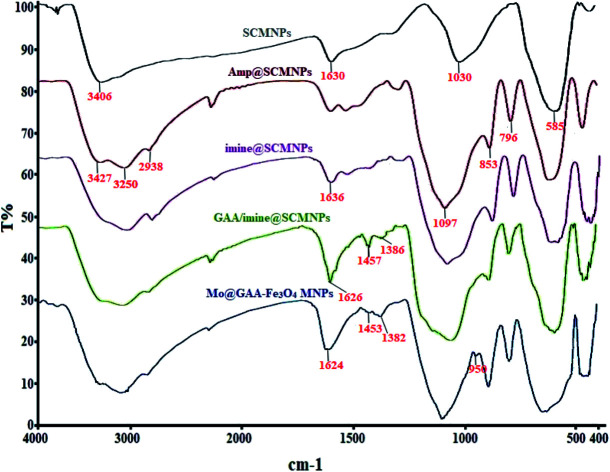

## 1. Introduction

In recent decades, the preparation of environmentally friendly catalysts with recoverability and reusability are the main challenges amongst researchers in organic chemistry. Chemical reactions can be accelerated in the presence of homogeneous or heterogeneous catalysts, each of which has its advantages and disadvantages. Homogeneous catalysts, due to the greater interaction with the substrates compared to their heterogeneous counterparts, can generate a uniform layer with the starting materials in the organic solvent of the reaction which increases their activity and selectivity during the corresponding reaction.^1–6^ However, the main problem when using these catalysts is related to the difficulty in separating and reusing them, which can be largely solved by using heterogeneous types. Heterogeneous catalysts, despite their lower efficiency, can exhibit suitable recyclability by centrifugation or filtration and can be reused several times without a significant decrease in their catalytic behavior. Functionalization of the heterogeneous supports *via* a strong connection with homogeneous catalysts increase their efficiency while maintaining their simplicity in recovery and separation.^7^

Nanocatalysts exhibit high catalytic activity, which is provided by their large surface area, and this significantly increases the contact between reactants and catalyst.^8,9^ Nowadays, remarkable attention has been paid to the use of magnetic nanoparticles (MNPs), as a bridge between homogeneous and heterogeneous catalysts, by scientific and industrial researchers because of their potential importance in modern chemical research.^10^ Some excellent physical and chemical features of MNPs such as separation by an external magnet, superparamagnetism, high surface area, and strong adsorption ability^11^ have expanded their utilization in the removal of heavy metal ions in wastewater,^12–14^ catalysis,^15^ and drug delivery.^16^ However, some of the MNPs features such as a tendency to oxidize and accumulate during reactions, high initial chemical activity, and high surface area to volume ratio will reduce their catalytic activity and magnetic nature during the reaction period.^17,18^ Thus, to overcome this drawback and increase their chemical stability for the special application, functionalization and modification of their surface with organic or inorganic supports are necessary.

Molybdenum as a d-block transition metal has various oxidation states from –I to +VI and it has a significant role in the life evolution. Despite the little amount of molybdenum on the earth's crust, it could be known as an oxotransferases for the active places and cofactors of several enzymes that catalyzes the oxygen and electron transfer reactions on the layers of nitrogen, carbon, and sulfur.^19,20^ Mo(vi) is one of the main oxidation states that obtains a formal double bond between the metal and the oxo ligand *via* the donating σ and π bonds. There is a wide range of reports on the fabricated complexes using dioxomolybdenum(vi) obtained from varying the type and denticity of the remaining anionic ligands.^21–24^ Among those, MoO_2_(acac)_2_ is one of the most famous and major dioxomolybdenum(vi) complexes used as a catalyst for the organic transformations including epoxidation of alkenes and the oxidation of sulfides.^25,26^ Also, the coordination of molybdenum complexes by the organic groups (including oxygen, nitrogen, and sulfur) stabilized on the surfaces of magnetic nanoparticles has been used in many catalytic systems.^27–30^

Multicomponent reactions (MCRs), as a versatile synthetic strategy, combine at least three or more reactants^31–34^*via* a single synthetic operation to generate several covalent bonds into structurally complex organic molecules containing most atoms of the available starting materials.^35^ Currently, MCRs are taken into consideration in the sustainable synthetic strategies, *via* which we can easily generate high compatibility with green chemistry due to their unique advantages, such as concomitant step economy, mild conditions, atom economy, and high convergence. Over the past decades and due to their various important applications in combinatorial chemistry,^36^ agrochemistry,^37^ medicinal chemistry,^38–40^ polymer chemistry,^31^ and natural product synthesis,^41^ MCRs have received considerable attention among organic chemists.

1,4-Dihydropyridine (1,4-DHP) core as an important class of nitrogen-containing heterocycles found in the nature have attracted considerable synthetic efforts over the past decade because of their diversity of biological functions, including antitubercular,^42^ neuroprotectant,^43^ antimicrobial,^44^ insecticidal,^45^ antiviral,^46^ antihypertension,^47^ anticancer,^48^ and antioxidant^49,50^ activities. Amongst them, polyhydroquinoline derivatives are very interesting heterocyclic molecules due to their pharmacological properties such as anti-inflammatory, antimalarial, antibacterial, anti-asthmatic, and tyrosine kinase inhibiting agents.^51–53^ Many procedures have been developed for the preparation of polyhydroquinolines using catalysts such as ionic liquids,^54^ microwaves,^55^ refluxing at high temperature,^56^ metal triflates,^57^ ceric ammonium nitrate (CAN),^58^ trimethylsilyl chloride (TMSCl),^59^ HY-zeolite,^60^ FeF_3_,^61^ silica perchloric acid (HClO_4_–SiO_2_),^62^ trifluoroethanol,^63^ montmorillonite K-10,^64^ iodine,^65^ autoclave,^66^ NiFe_2_O_4_ MNPs,^67^ heteropoly acid,^68^l-proline,^69^ PTSA-SDS,^70^ NiCuMgFe_2_O_4_ MNPs,^71^ polymers,^72^ Sc(OTf)_3_,^73^ {Fe_3_O_4_@SiO_2_@(CH_2_)_3_Im}C(NO_2_)_3_,^74^ Yb(OTf)_3_,^57^ Fe_3_O_4_-adenine-Ni,^75^ BINOL-phosphoric acid derivatives,^76^ and Cu-SPATB/Fe_3_O_4_.^77^ Although most of these procedures offer distinct merits, some of these procedures suffer from one or more limitations, such as generating a large amount of waste, low yields of the desired product, poor recovery of the catalyst, long reaction times, and hard reaction conditions. Therefore, to avoid these limitation based on the green chemistry protocols, the discovery of simple, efficient, versatile, and environmentally friendly processes for the synthesis of polyhydroquinolines is still favored.

## 2. Experimental

All of the chemical substances used in this work were purchased from Merck, Aldrich, and Fluka Chemical Companies and used without further purification. Melting points of the products were determined with an Electrothermal 9100 apparatus. Energy-dispersive X-ray spectroscopy (EDX) was carried out on a FE-SEM (MIRA III, Detector of SAMX, France). Powder X-ray diffraction (XRD) was performed using Cu-Kα radiation (*λ* = 1.54 Å) on a Philips-PW1730 in the 2*θ* range of 10°–80°. Thermogravimetric analyses (TGA) were performed using a Linseis SATPT 100 thermoanalyzer at a heating rate of 10 °C min^−1^ under nitrogen atmosphere over a temperature range of 25–700 °C. The Fourier-transform infrared (FT-IR) spectra were recorded on a Perkin Elmer PXI spectrum, using pellets of the materials diluted with KBr in the range of 400–4000 cm^−1^. The magnetic features of the catalyst were determined using a vibrating sample magnetometer (VSM; MDK Co. Kashan, Iran) in the magnetic field range of −15 000 Oe to 15 000 Oe at room temperature. Scanning electron microscopy (SEM) images were recorded using an SEM-LEO 1430VP analyzer. Transmission electron microscopy (TEM) was utilized on a Zeiss-EM 900 instrument.

### 2.1. Synthesis of Fe_3_O_4_ nanoparticles (MNPs)

In the first step, a solution of FeCl_2_·4H_2_O (0.86 g) and FeCl_3_·6H_2_O (2.36 g) were dissolved in deionized water (40 mL). The mixture was heated to 90 °C, and mechanical stirring was done for 30 min under an argon atmosphere. Then, 10 mL of ammonia solution (25%) was added dropwise to the resulting mixture and stirred for another 20 min under argon flow. The precipitates were washed with distilled water and collected using a magnet. The product was dried under vacuum conditions.

### 2.2. Synthesis of Fe_3_O_4_@SiO_2_ (SCMNPs)

In a typical method, 1.0 g of obtained Fe_3_O_4_ nanoparticles was dispersed in a mixture of 60 mL ethanol, 20 mL deionized water, and 2 mL ammonia solution (25%) by sonication for 10 min. Then, 0.45 mL of tetraethylorthosilicate (TEOS) was added into the reaction system, sonicated for another 10 min, and stirred at ambient temperature for 14 h. The resultant precipitate was magnetically collected by a permanent magnetic field, washed three times with a mixture of ethanol and water (1 : 1), and dried in a vacuum oven.

### 2.3. Functionalization of SCMNPs by 3-aminopropyltriethoxysilane (Amp@SCMNPs)

1 g of core–shell Fe_3_O_4_@SiO_2_ nanoparticles was suspended in 20 mL of dry toluene by ultrasonication. After treatment for 20 min, 2 mL of 3-aminopropyltriethoxysilane (Amp) was added to the solution and refluxed under an argon atmosphere. After 24 h, the Amp@SCMNPs were collected using a permanent magnet, washed with ethanol and distilled water several times, and then dried under vacuum.

### 2.4. Functionalization of Amp@SCMNPs by glutaraldehyde (imine@SCMNPs)

2 g of prepared Amp@SCMNPs nanoparticles was added to 100 mL ethanol and dispersed for 30 min under ultrasonic irradiation. Then, 4 mmol of glutaraldehyde was added into the reaction solution and refluxed for 24 h. The prepared solid product (imine@SCMNPs) was then magnetically isolated and washed several times with ethanol to remove unreacted glutaraldehyde; finally, it was dried under vacuum.

### 2.5. Synthesis of GAA/imine@SCMNPs

1 g of the obtained imine@SCMNPs was dispersed in 25 mL DMF by the ultrasonic bath for 30 min. Then, 0.85 mmol of guanidineacetic acid (GAA) was added to the reaction solution. After the stirrer process for 8 h, the resulting substance was separated by a permanent magnet, rinsed with ethanol several times, and dried under vacuum.

### 2.6. Synthesis of Mo@GAA-Fe_3_O_4_ MNPs

**Scheme 1:**
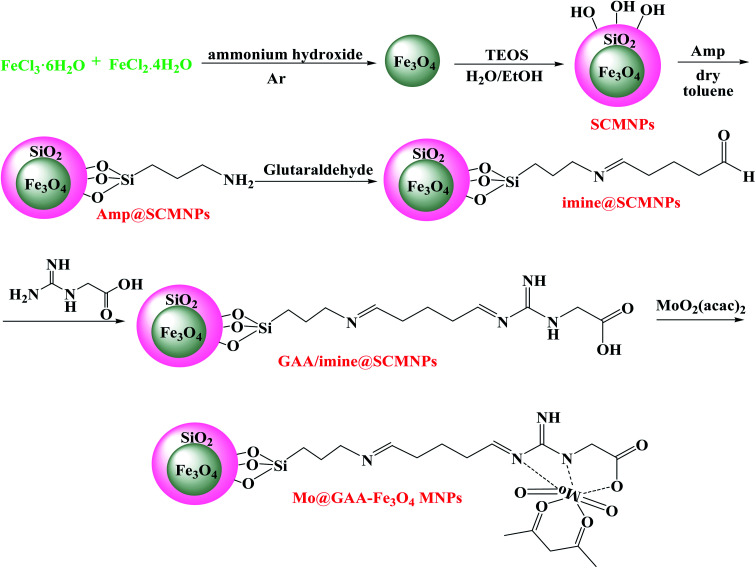
The Mo@GAA-Fe_3_O_4_ MNPs synthesis.

MoO_2_(acac)_2_ was prepared using the literature method.^78^ 2 g of GAA/imine@SCMNPs were added into 150 mL ethanol and a solution of MoO_2_(acac)_2_ (4 mmol) in 70 mL ethanol was added to this reaction solution and refluxed for 12 h. After magnetic separation, the resulting solid product was washed with dichloromethane to remove the unreacted MoO_2_(acac)_2_ and was dried under vacuum (Scheme 1).

### 2.7. General procedure for the preparation of polyhydroquinolines

Firstly, Mo@GAA-Fe_3_O_4_ MNPs (10 mg) was poured into the reaction mixture of dimedone or 1,3-cyclohexandione (1 mmol), ethyl acetoacetate, or methyl acetoacetate (1 mmol), aldehydes (1 mmol), and ammonium acetate (1.2 mmol), and the reaction mixture was heated for a specific time. Upon completion of the reaction, the catalyst was easily extracted using an external magnetic field; the resulting product was collected by filtration, rinsed, and recrystallized with ethanol to give pure polyhydroquinolines.

## 3. Results and discussion

### 3.1. FTIR analysis Mo@GAA-Fe_3_O_4_ MNPs

**Fig. 1:**
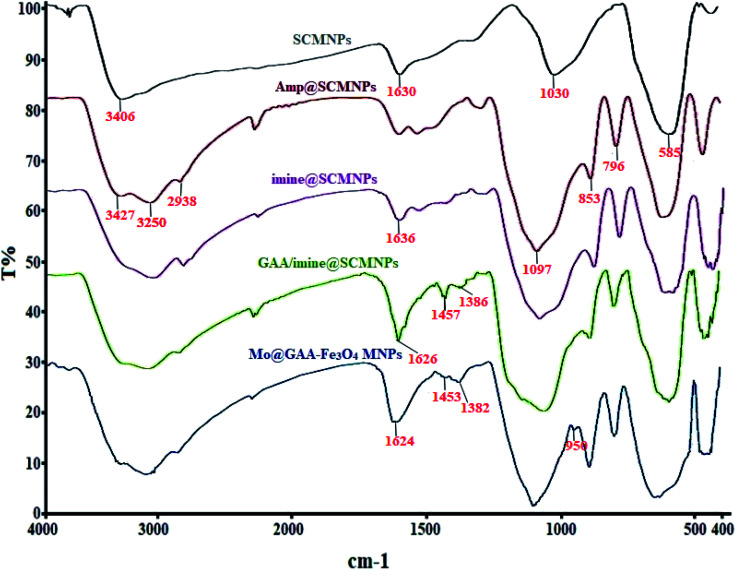
FTIR spectra of SCMNPs, Amp@SCMNPs, imine@SCMNPs, GAA/imine@SCMNPs, and Mo@GAA-Fe_3_O_4_ MNPs.

FTIR spectroscopy is one of the main techniques for the qualitative determination of the molecular structures in different species and the identification of existing functional groups in the structure. Fig. 1 shows FTIR spectra for SCMNPs, Amp@SCMNPs, imine@SCMNPs, GAA/imine@SCMNPs, and Mo@GAA-Fe_3_O_4_ MNPs. In the case of SCMNPs, the band at 585 cm^−1^, from the Fe–O bond, verifies that the Fe_3_O_4_ lattice is formed. The presence of a broad peak at 3406 cm^−1^ is due to the O–H stretching vibrations, which are attached to the Fe_3_O_4_ surface. The above-mentioned structure has the symmetric stretching vibrations of Si–O–Si at around 1030 cm^−1^ and twisting bonds of Si–O–H and H–O–H at 1630 cm^−1^, indicating that the Fe_3_O_4_ nanoparticles (MNPs) contain silica layers. In the FT-IR spectrum of the Amp@SCMNPs can be observed the peaks for C–H (2938 cm^−1^), and NH_2_ (3250, and 3427 cm^−1^) stretching vibrations, which confirmed the successful covalent attachment of 3-aminopropyltriethoxysilane (Amp) to the SCMNPs surface. Also, vibrations at 796 and 853 cm^−1^ were probably attributed to the asymmetric stretching and in plane bending of Si–O–Si group, respectively. In about the imine@SCMNPs, the peak at 1636 cm^−1^ is assigned to the stretching vibrations of the C=N bond, revealing the functionalization of the Amp@SCMNPs with glutaraldehyde groups. The bands at 1457 and 1626 cm^−1^ of the FTIR spectrum of GAA/imine@SCMNPs can be assigned to the C–N and COO stretching vibrations. Also, a broad band about 3000–3400 cm^−1^ can be ascribed to the acidic OH stretching vibrations. The peak observed at 950 cm^−1^ corresponds to the MoO_2_ group, which indicates the formation of the Mo@GAA-Fe_3_O_4_ MNPs. Moreover, the FTIR spectra of the catalyst show a frequency shift for some bonds, indicating the coordination of the metal with the desired bonds.

### 3.2. TGA analysis of Mo@GAA-Fe_3_O_4_ MNPs

**Fig. 2:**
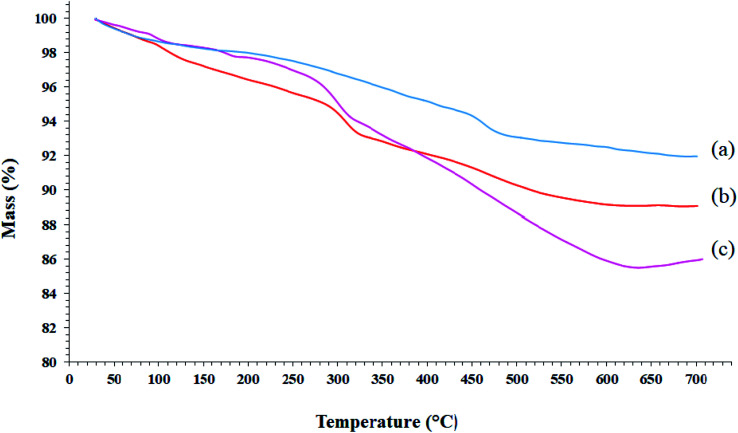
TGA curves of (a) SCMNPs, (b) GAA/imine@SCMNPs, and (c) Mo@GAA-Fe_3_O_4_ MNPs.

TGA analysis can be used to determine the loading amount of the organic groups on the synthetic substrates. This measures the mass variations as a function of the temperature and time under a controlled atmosphere and indicates the thermal stability of the sample on a plot. The TGA analysis curves of SCMNPs, GAA/imine@SCMNPs, and Mo@GAA-Fe_3_O_4_ MNPs are shown in Fig. 2. All samples underwent a small weight loss below 200 °C due to water thermodesorption from the surface (drying). Another weight loss up to 700 °C was found in TGA curve of SCMNPs, which can be related to the release of hydroxyl ions from the nanoparticles and volatilization. In the TGA curves of GAA/imine@SCMNPs and Mo@GAA-Fe_3_O_4_ MNPs, another weight loss can be seen up to 650 °C, which can be attributed to the decomposition of the functionalized organic groups.

### 3.3. XRD analysis of Mo@GAA-Fe_3_O_4_ MNPs

**Fig. 3:**
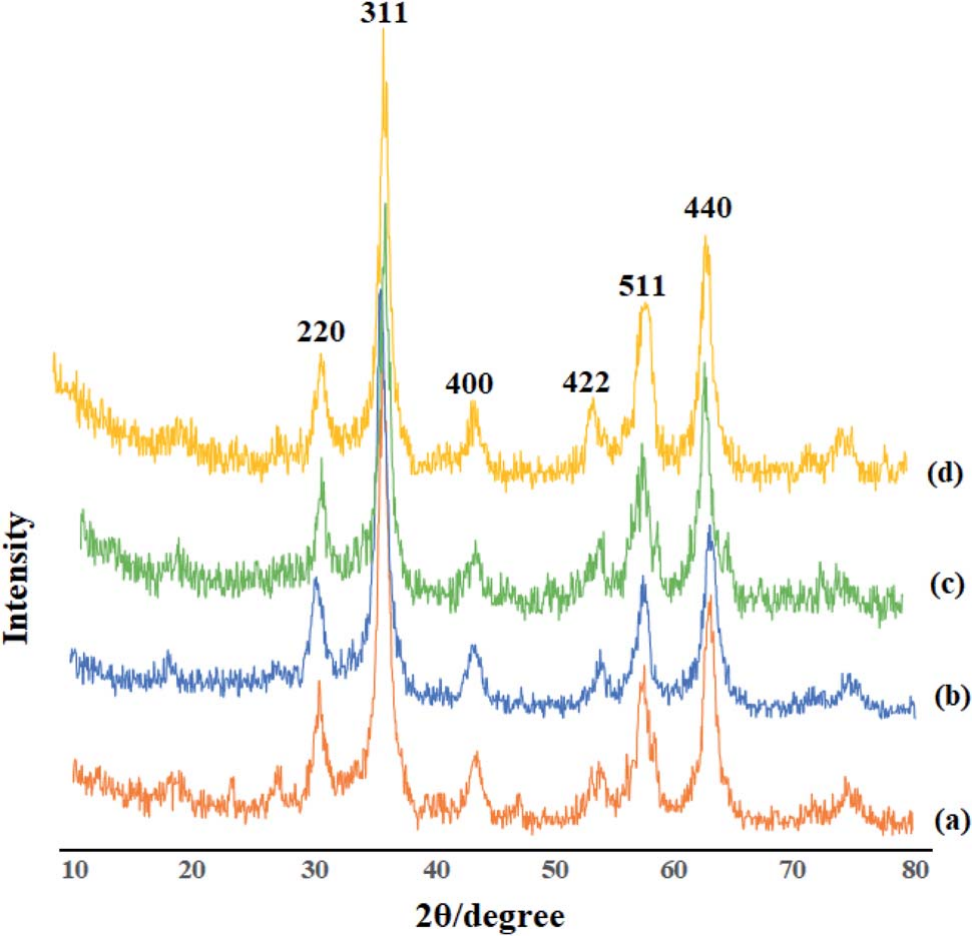
XRD patterns of (a) Fe_3_O_4_, (b) GAA/imine@SCMNPs, (c) Mo@GAA-Fe_3_O_4_ MNPs, and recovered Mo@GAA-Fe_3_O_4_ MNPs (d).

Identification of the crystalline materials using X-ray is one of the most common and important analysis techniques. As the X-ray region is located between gamma and ultraviolet, useful data could be obtained from this spectral region including the crystalline structure, the material type, and the nanoparticle size. The XRD patterns for Fe_3_O_4_ (a), GAA/imine@SCMNPs (b), Mo@GAA-Fe_3_O_4_ MNPs (c) and recovered Mo@GAA-Fe_3_O_4_ MNPs (d) are depicted in Fig. 3. The X-ray diffraction patterns of Fe_3_O_4_ magnetic nanoparticles display six diffraction peaks at 2*θ* = 30.5°, 35.7°, 43.8°, 53.6°, 57.5°, and 63.1° (indexed as (220), (311), (400), (422), (511), and (440) reflection, associated with the crystalline structure of the spinel magnetic core). In addition, GAA/imine@SCMNPs and Mo@GAA-Fe_3_O_4_ MNPs have also similar diffraction peaks, indicating an unchanged crystalline structure of the above-mentioned products. It is necessary to mention that the XRD pattern of recovered Mo@GAA-Fe_3_O_4_ MNPs after the first recovery and reuse do not show any change in crystalline structure.

### 3.4. VSM analysis of Mo@GAA-Fe_3_O_4_ MNPs

**Fig. 4:**
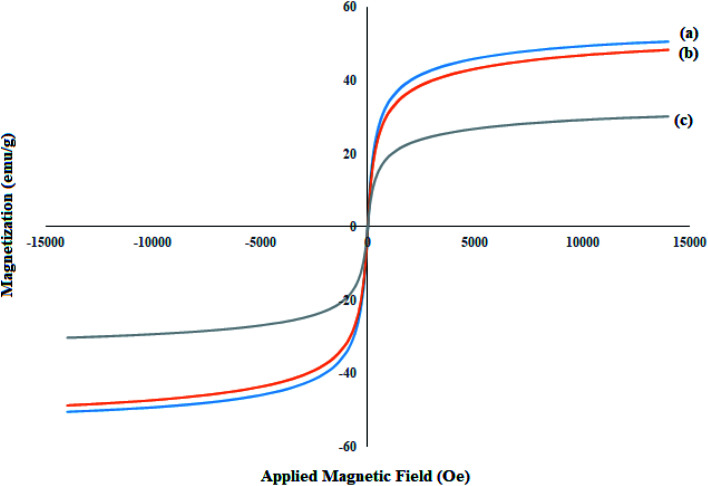
VSM patterns of (a) Fe_3_O_4_, (b) SCMNPs, and (c) Mo@GAA-Fe_3_O_4_ MNPs.

Magnetic properties and the behavior of the achieved nanoparticles are investigated by the VSM analysis. An external magnetic field is applied to evaluate the magnetization ability of the magnetic nanoparticles. The VSM analysis of the synthesized (a) Fe_3_O_4_, (b) SCMNPs, and (c) Mo@GAA-Fe_3_O_4_ MNPs were shown in Fig. 4. The saturation magnetization values (*M*_s_) of magnetic materials revealed significant differences, which are 50.63 and 47.16 emu g^−1^ for bare Fe_3_O_4_ and SCMNPs, respectively, while for catalyst, the difference is 28.99 emu g^−1^. As can be seen, the value of *M*_s_ for the catalyst decreased, which can be attributed to the functionalization of the Fe_3_O_4_ core by silica layers, organic molecules, and metal groups.

### 3.5. SEM analysis of Mo@GAA-Fe_3_O_4_ MNPs

**Fig. 5:**
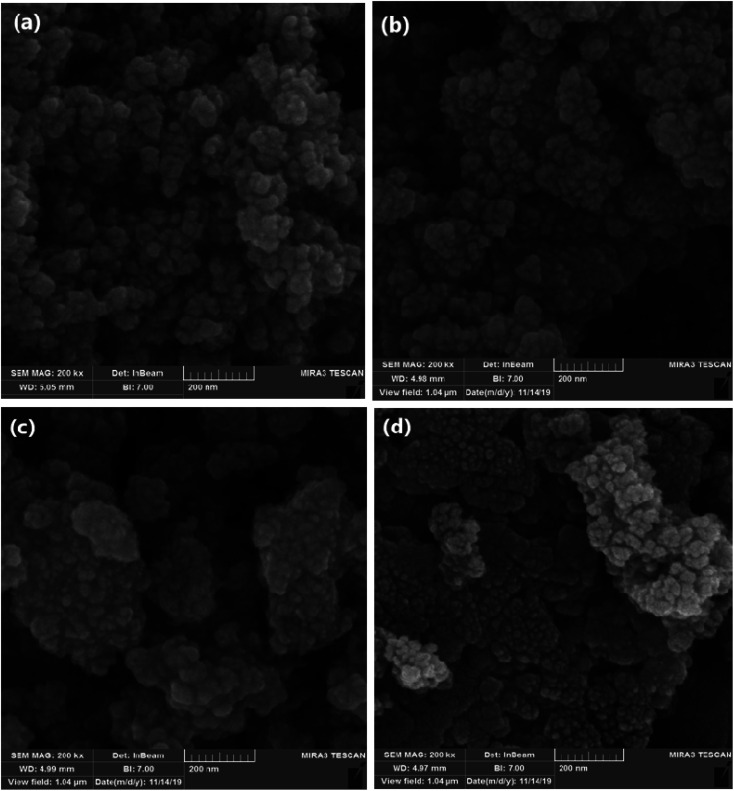
SEM image of (a) Fe_3_O_4_, (b) SCMNPs, (c) GAA/imine@SCMNPs, and (d) Mo@GAA-Fe_3_O_4_ MNPs.

The obtained images from the SEM technique could be used to study the morphology, uniformity, and physical properties of nanoparticle surfaces. High magnifications are obtained by this device to precisely investigate the material details. As shown in Fig. 5, Fe_3_O_4_ and SCMNPs are nearly spherical with a smooth surface and their mean particle sizes are 19 and 26 nm, respectively. The obtained results clearly showed that the SCMNPs particles have grown compared to the Fe_3_O_4_. According to the represented images for GAA/imine@SCMNPs and Mo@GAA-Fe_3_O_4_ MNPs, the average size of the above-mentioned nanoparticles are in the range of 30–37 nm. The modification by organic and metallic groups can result in the growth and accumulation of the nanoparticles.

### 3.6. TEM analysis of Mo@GAA-Fe_3_O_4_ MNPs

**Fig. 6:**
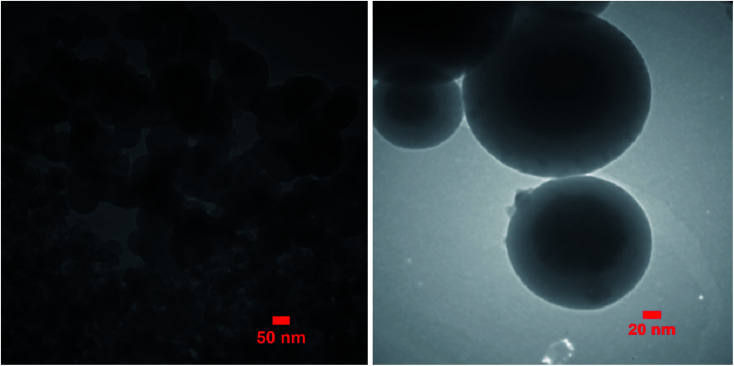
TEM image of Mo@GAA-Fe_3_O_4_ MNPs.

In TEM analysis, a focused electron beam is used for obtaining the images. In this technique, some information from the inner structure could be obtained by transmitting a high-energy electron beam through a thin sample. Fig. 6 shows the TEM image of Mo@GAA-Fe_3_O_4_ MNPs. TEM images indicate that the diameter of the obtained catalyst is about 28–35 nm and it has a nearly spherical morphology with a narrow particle size distribution.

### 3.7. EDX and elemental mapping analysis of Mo@GAA-Fe_3_O_4_ MNPs

**Fig. 7:**
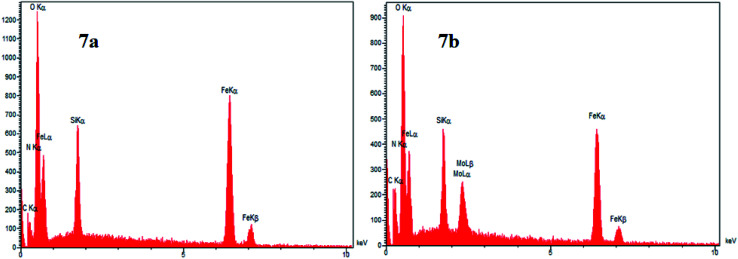
EDX spectra of (a) GAA/imine@SCMNPs and (b) and Mo@GAA-Fe_3_O_4_ MNPs.

**Fig. 8:**
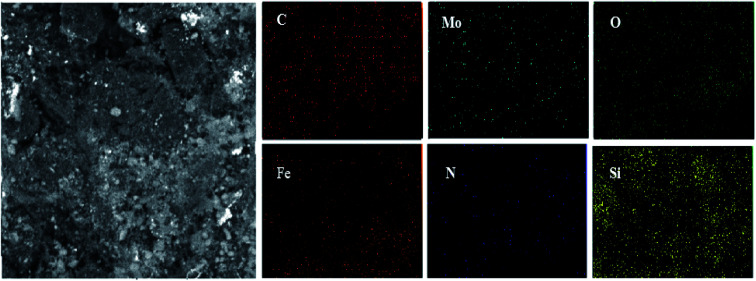
SEM image of Mo@GAA-Fe_3_O_4_ MNPs nanocatalyst and corresponding quantitative EDX element mapping of C, N, O, Fe, Si, and Mo.

One of the main techniques to identify the elemental composition of the sample or a part of it is the EDX analysis. The EDX spectra of the GAA/imine@SCMNPs and Mo@GAA-Fe_3_O_4_ MNPs is shown in Fig. 7a and b, respectively. In the case of GAA/imine@SCMNPs, the presence of carbon, nitrogen, oxygen, iron, and silicon was confirmed (Fig. 7a). Also in Fig. 7b, the presence of the molybdenum element indicates coordination of Mo with nitrogen and oxygen electron pairs and thus the successful synthesis of the Mo@GAA-Fe_3_O_4_ MNPs. In addition, Fig. 8 shows the elemental map of the Mo@GAA-Fe_3_O_4_ MNPs nanocatalyst, which exhibits the presence of C, N, O, Fe, Si, and Mo elements.

**Scheme 2:**
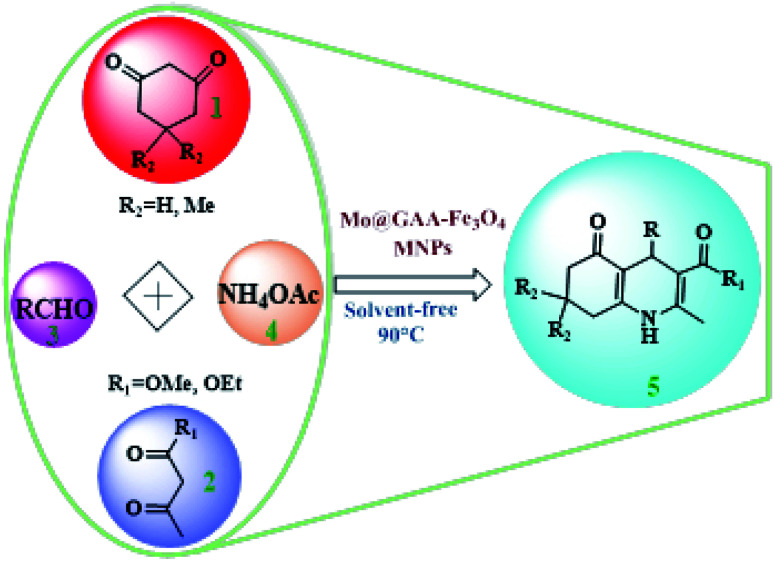
Mo@GAA-Fe_3_O_4_ MNPs-catalyzed synthesis of polyhydroquinolines.

To synthesize new catalysts for certain organic processes,^79–84^ the synthesis of potentially active polyhydroquinoline derivatives (5) was investigated with various substituents from the reaction between dimedone or 1,3-cyclohexandione (1), ethyl acetoacetate, or methyl acetoacetate (2), aldehydes (3), and ammonium acetate (4) under solvent-free conditions using Mo@GAA-Fe_3_O_4_ MNPs as a novel, eco-friendly, reusable, and promising nanocatalyst (Scheme 2).

The catalytic activity of the Mo@GAA-Fe_3_O_4_ MNPs was surveyed in the preparation of polyhydroquinoline derivatives. Firstly, the condensation reaction of dimedone, ethyl acetoacetate, 4-chlorobenzaldehyde, and ammonium acetate in the presence of Mo@GAA-Fe_3_O_4_ MNPs was chosen as a model reaction. To optimize the reaction conditions, the synthesis of polyhydroquinolines we studied under various reaction conditions, including solvent, temperature, and the amount of catalyst. To optimize the reaction solvent, H_2_O, EtOH, THF, CH_2_Cl_2_, CH_3_CN, toluene, cyclohexane, and solvent-free conditions were tested (Entries 1–8). The results are summarized in Table 1, showing that carrying out the reaction in the absence of solvent led to the formation of the desired product 5p in the highest yield (Entry 8). However, a good yield of the product was obtained in EtOH after 50 min from the start of the reaction (Entry 2). To study the role of catalyst amount, the model reaction was performed in the presence of 5, 10, 15, and 20 mg of Mo@GAA-Fe_3_O_4_ MNPs (Entries 8 and 10–12). These observations showed that carrying out the reaction in the absence of the catalyst gave any yield for the desired product (Entry 9). The reaction in presence of 5 mg of the catalyst provided a good yield of the product (Entry 10), while 10, 15, and 20 mg gave excellent-to-high yields of the corresponding product (Entries 8 and 11–12). It was found that using 10 mg of the Mo@GAA-Fe_3_O_4_ MNPs is appropriate to carry out the reaction under solvent-free conditions with a 96% yield. Afterward, the influence of temperature on the model reaction was investigated. The reaction was carried out under different temperatures (25, 40, 60, 80, 90, and 100 °C) (Entries 8 and 13–17), and the best result was achieved at 90 °C under solvent-free conditions (Entry 8). Finally, when the model reaction was done in the existence of 10 mg of imine@SCMNPs, GAA/imine@SCMNPs under the optimized conditions, the product yield were 86 and 88%, respectively (Entries 18–19). Comparing the product efficiencies for the inputs of 8 and 18–19, it could be fully understood that the coordination of the compound Mo@GAA-Fe_3_O_4_ MNPs by free electron pair of nitrogen with the molybdenum complex increased the catalytic activity.

**Table 1:** Optimization one-pot four-component condensation of dimedone, ethyl acetoacetate, 4-chlorobenzaldehyde, and ammonium acetate, under different conditions^a^

Entry	Solvent	Catalyst (mg)	Temp. (°C)	Time (min)	Yield^b^ (%)
1	H_2_O	10	Reflux	100	45
2	EtOH	10	Reflux	50	83
3	THF	10	Reflux	160	18
4	CH_2_Cl_2_	10	Reflux	360	25
5	CH_3_CN	10	Reflux	220	35
6	Toluene	10	Reflux	360	39
7	Cyclohexane	10	Reflux	200	40
8	—	10	90	12	96
9	—	—	90	100	—
10	—	5	90	35	81
11	—	15	90	12	95
12	—	20	90	12	92
13	—	10	100	12	95
14	—	10	80	20	88
15	—	10	60	40	76
16	—	10	40	90	65
17	—	10	25	120	21
18	—	10 (imine@SCMNPs)	90	25	86
19	—	10 (GAA/imine@SCMNPs)	90	25	88

a Reaction conditions: dimedone (1 mmol), ethyl acetoacetate (1 mmol), 4-chlorobenzaldehyde (1 mmol), and ammonium acetate (1.2 mmol) and required amount of the catalyst.

b Isolated yield.

Utilizing the optimal reaction conditions, various polyhydroquinolines were prepared by the four-component reaction of dimedone, or 1,3-cyclohexandione, ethyl acetoacetate or methyl acetoacetate, a wide range of aldehydes, and ammonium acetate (Table 2). Aromatic aldehydes with electron-donating or electron-withdrawing groups tolerate smooth transformation to the corresponding products without the formation of by-products at better yields and in short reaction times. The reaction was also carried out with aliphatic aldehyde instead of aromatic aldehyde but did not afford the title products in considerable amounts (5y).

**Table 2:** Preparation of polyhydroquinoline derivatives using desired catalyst^a^

Entry	RCHO (3)	Product	Time (min)	Yield^b^ (%)	M.P. (Obsd) (°C)	M.P. (Lit) (°C)
1	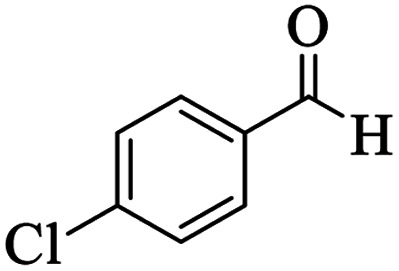	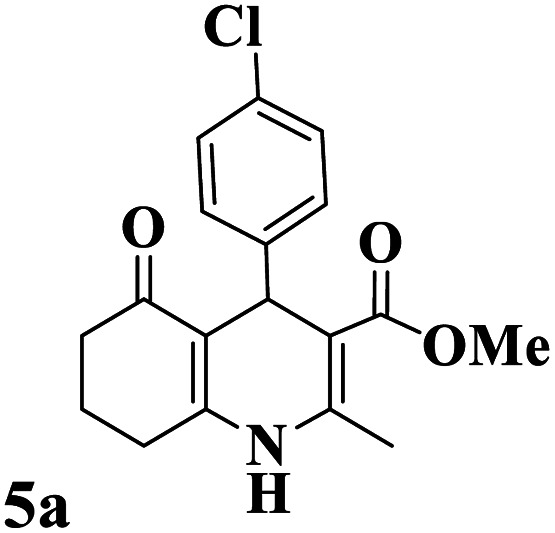	13	93	222–224	221–223 (ref. 85)
2	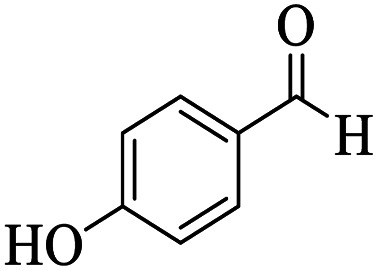	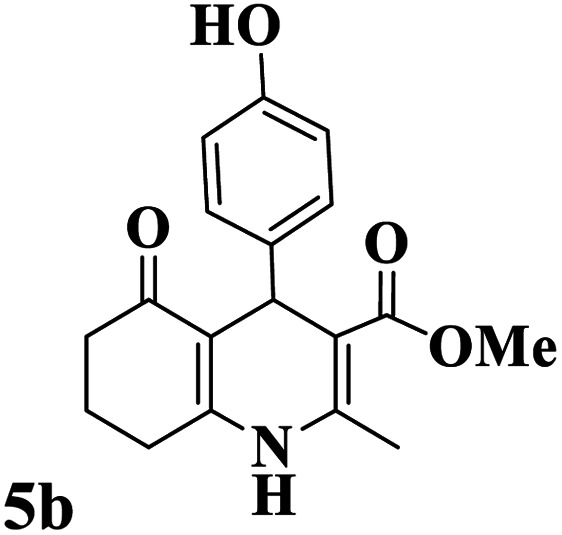	15	93	253–256	256–257 (ref. 85)
3	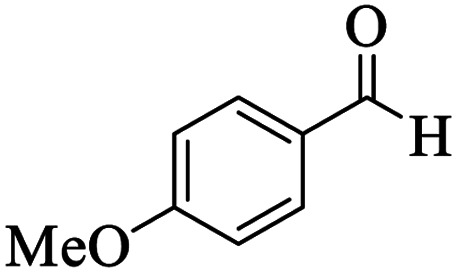	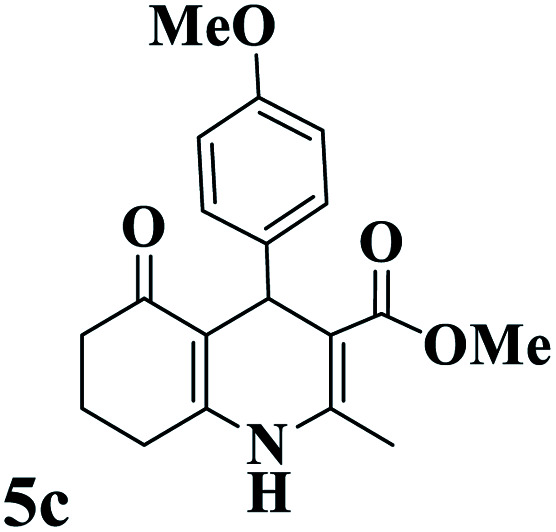	20	92	208–211	208–211 (ref. 86)
4	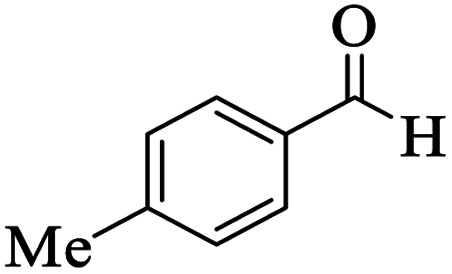	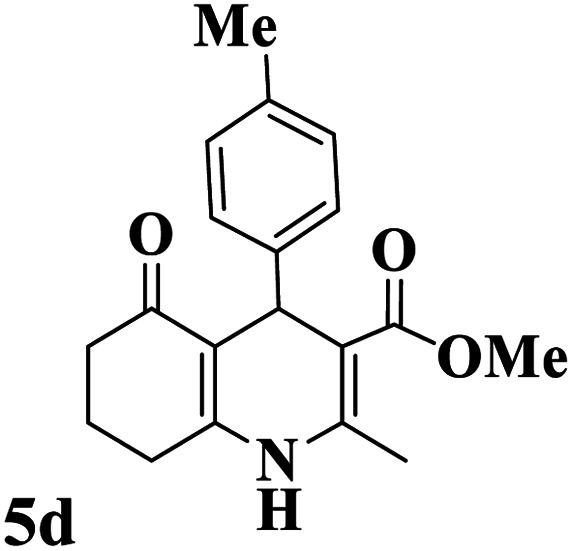	17	90	236–238	238–240 (ref. 87)
5	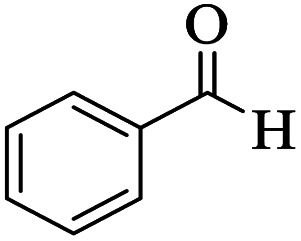	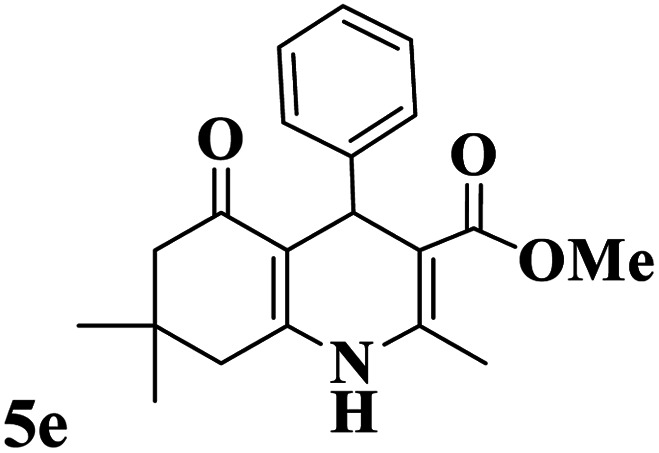	13	93	257–259	257–259 (ref. 88)
6	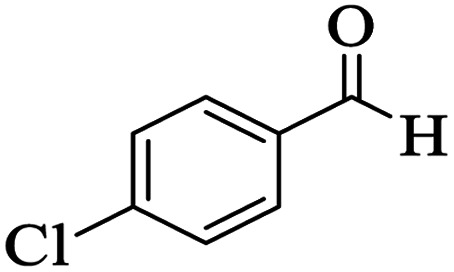	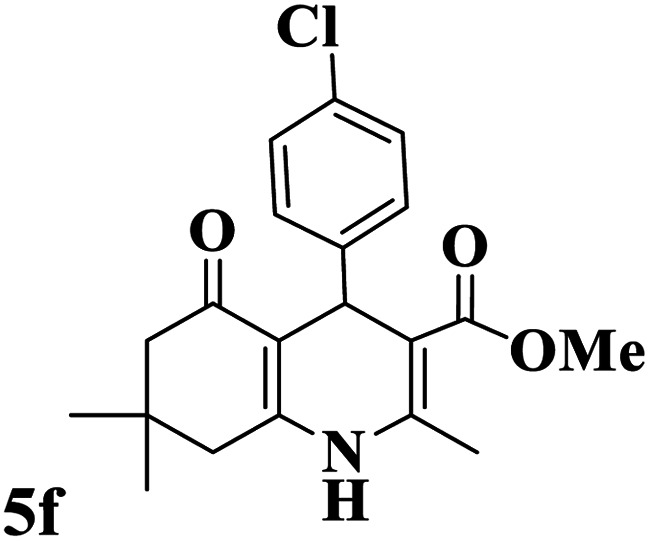	15	93	219–221	220–222 (ref. 89)
7	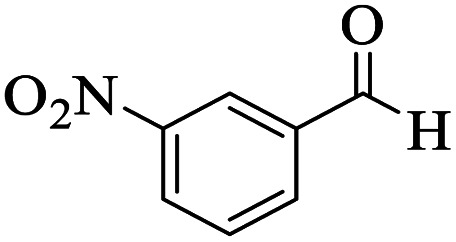	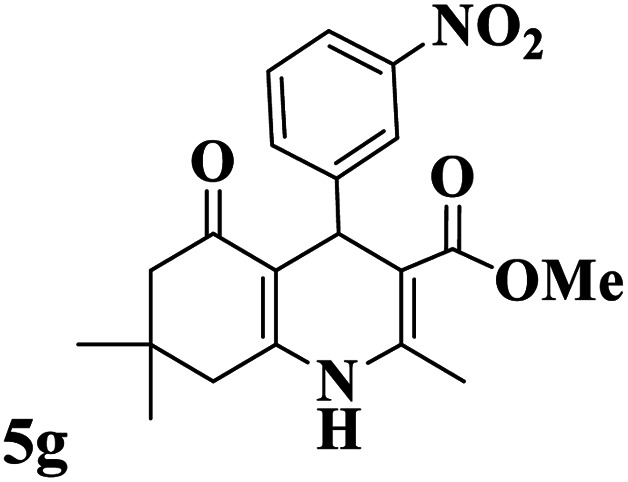	20	90	223–225	222–223 (ref. 90)
8	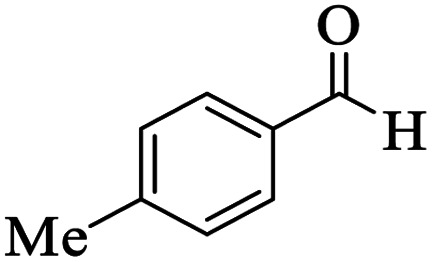	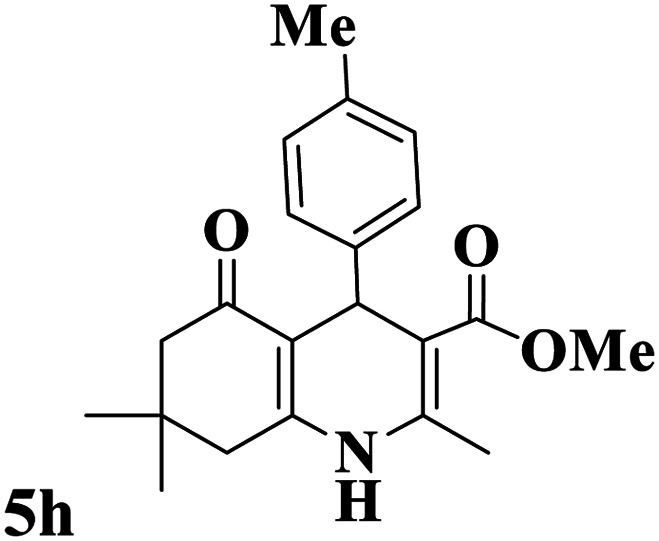	20	93	269–272	270–274 (ref. 91)
9	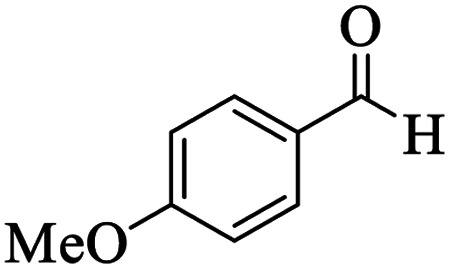	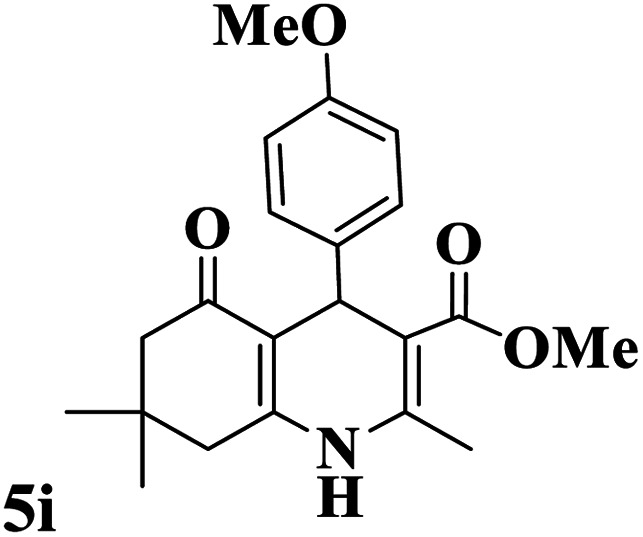	15	95	256–258	256–259 (ref. 88)
10	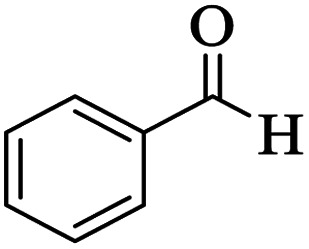	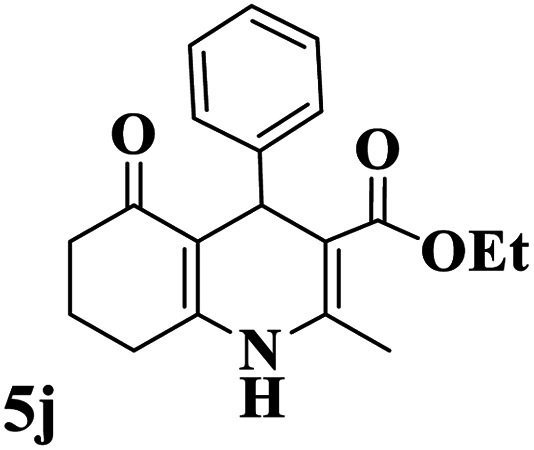	14	94	239–241	240–241 (ref. 91)
11	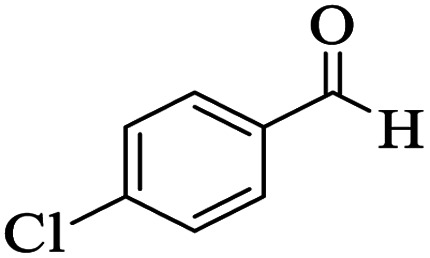	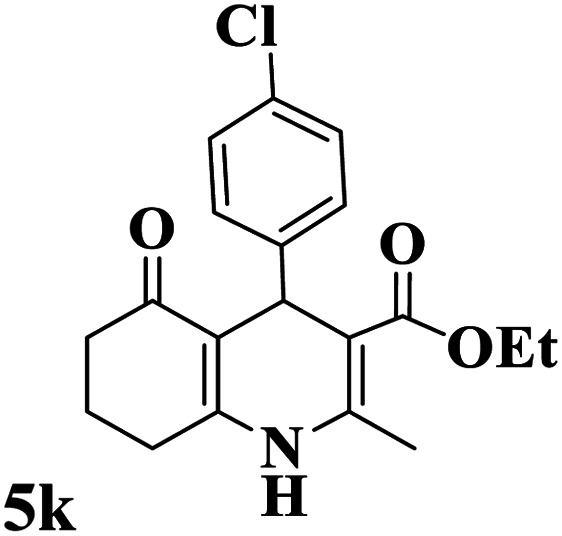	15	94	234–236	234–235 (ref. 91)
12	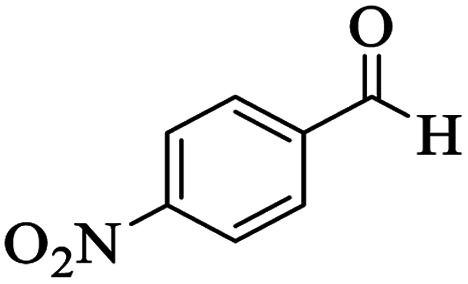	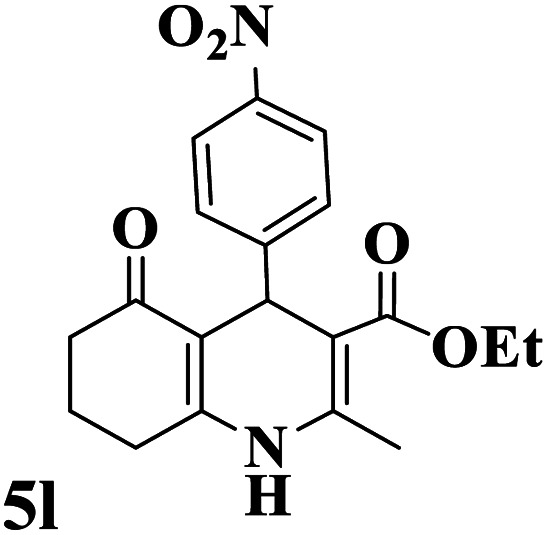	18	91	202–205	204–205 (ref. 91)
13	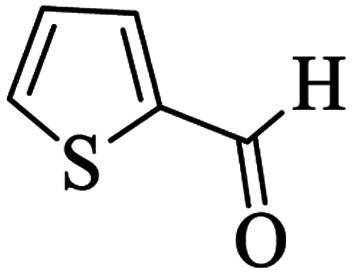	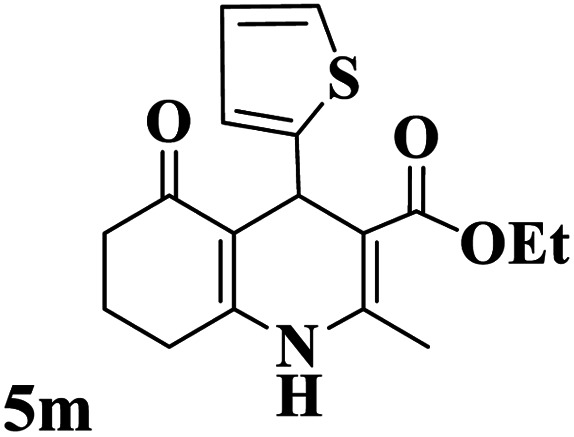	20	88	231–235	233–234 (ref. 91)
14	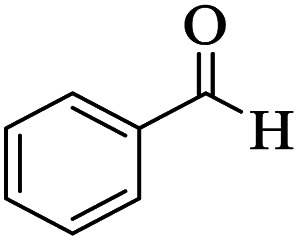	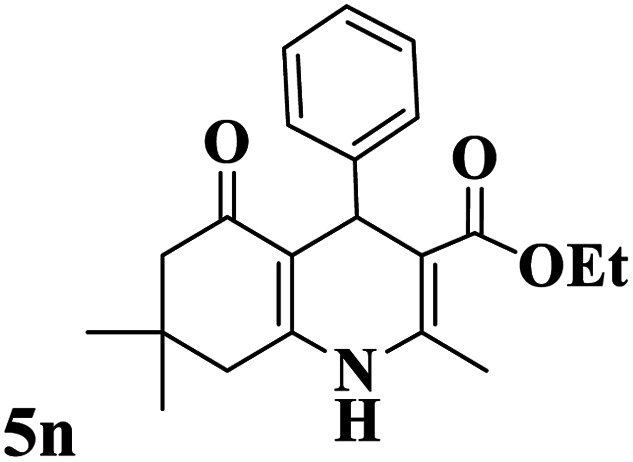	13	95	201–203	202–204 (ref. 57)
15	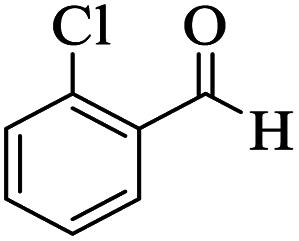	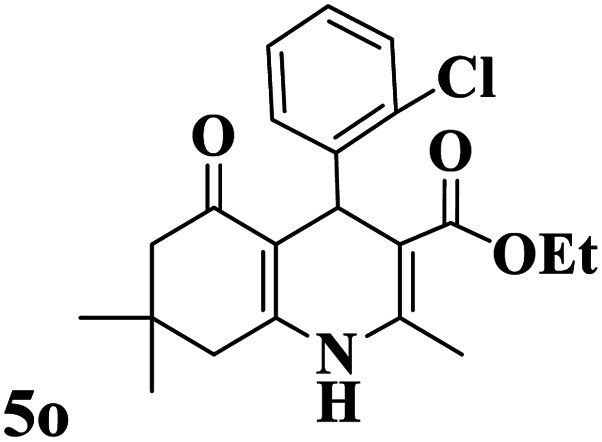	15	90	207–208	206–208 (ref. 92)
16	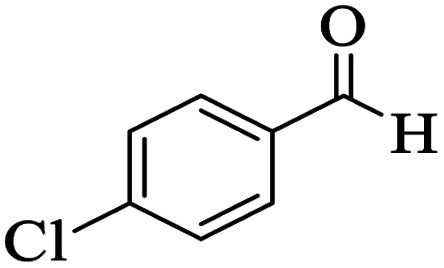	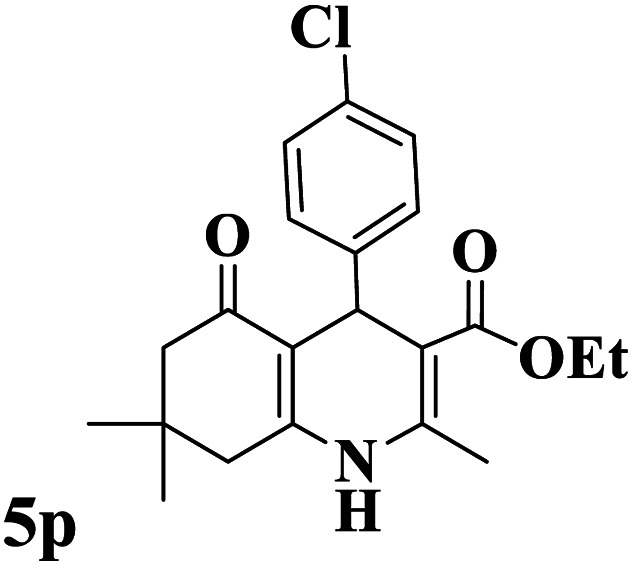	12	96	243–245	244–246 (ref. 93)
17	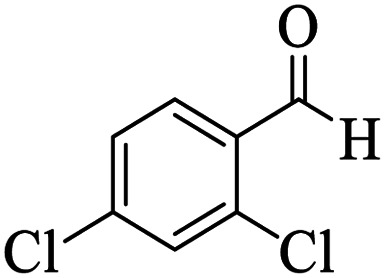	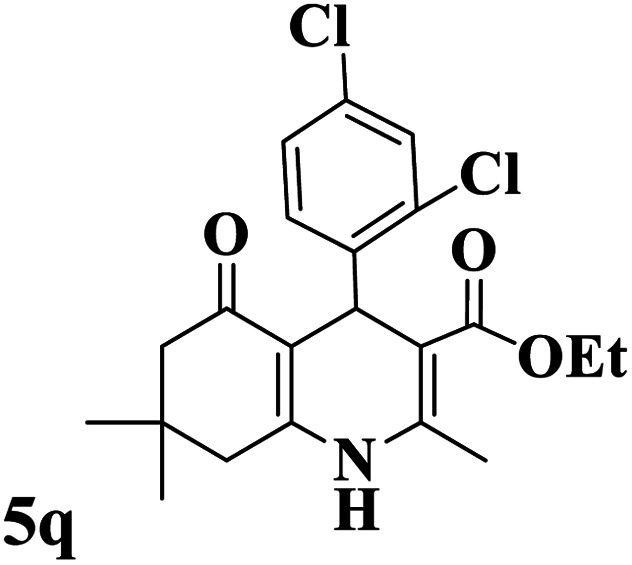	14	92	240–242	241–243 (ref. 57)
18	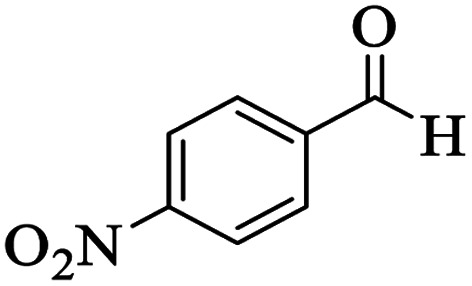	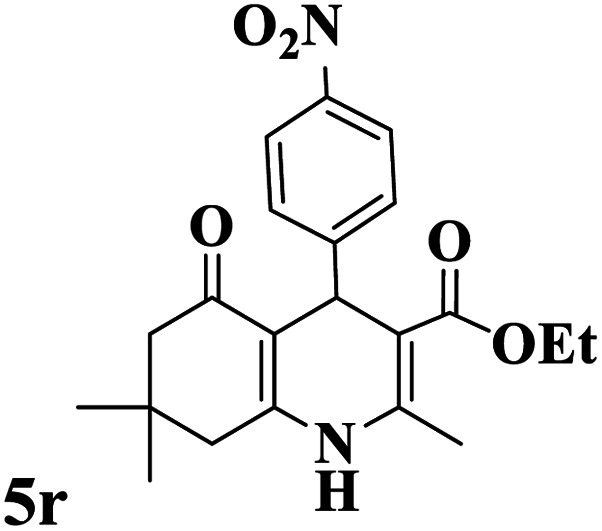	14	89	245–247	244–246 (ref. 92)
19	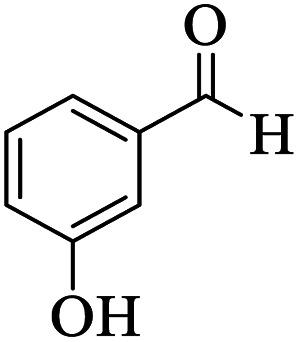	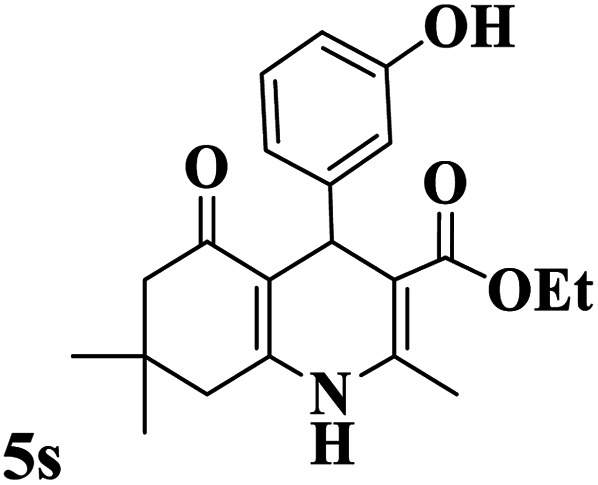	15	91	218–221	218–220 (ref. 92)
20	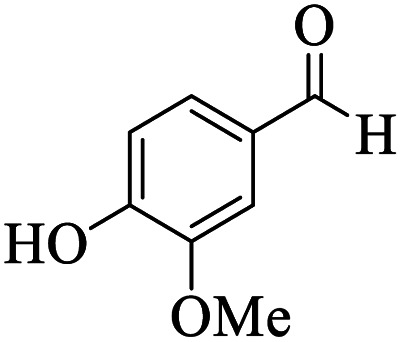	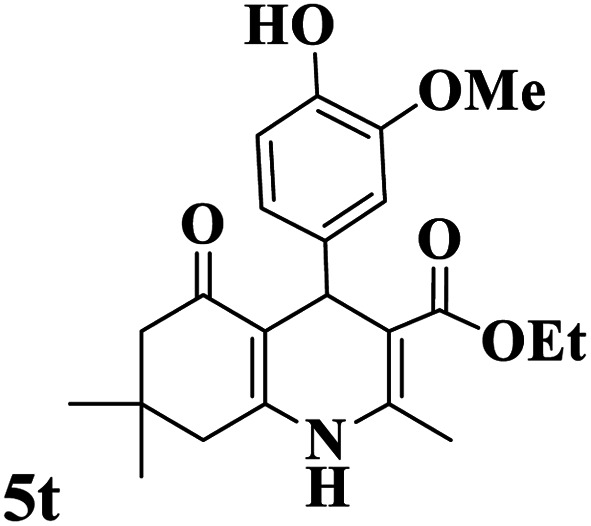	20	92	210–213	211–212 (ref. 72)
21	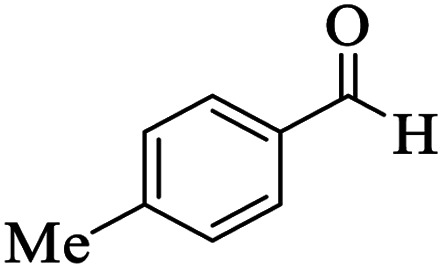	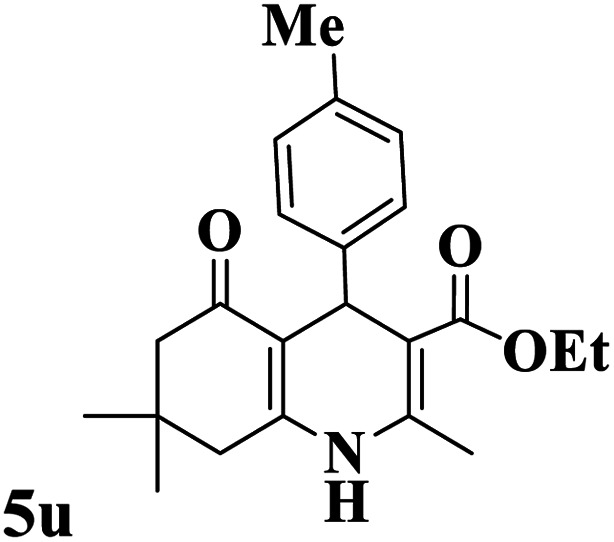	18	89	259–262	260–261 (ref. 57)
22	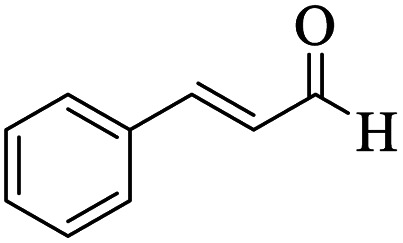	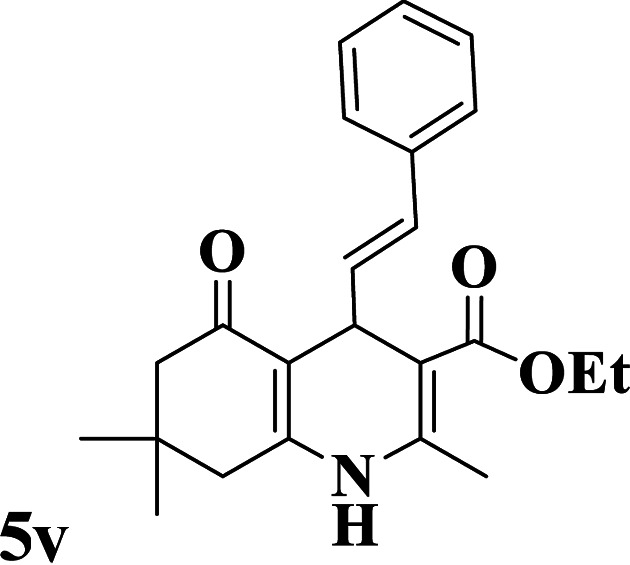	18	94	205–207	204–206 (ref. 57)
23	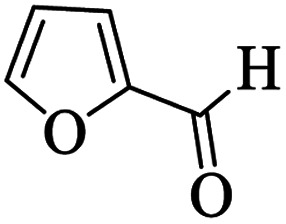	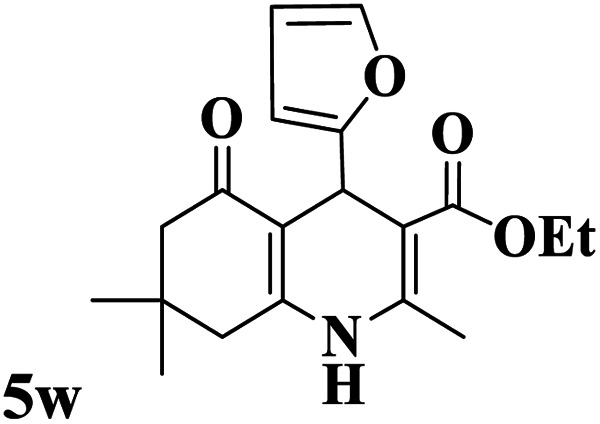	20	90	247–249	246–248 (ref. 57)
24	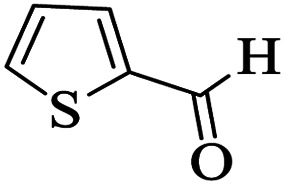	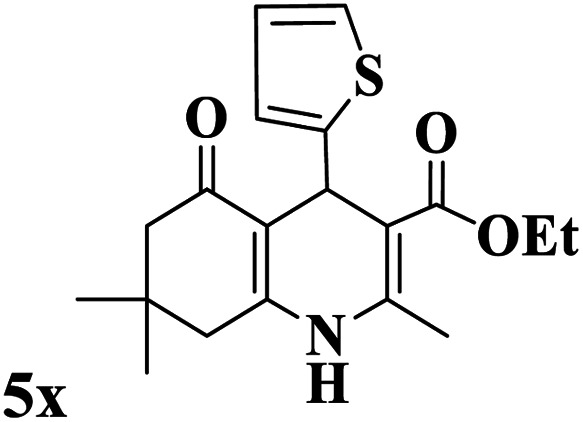	20	88	237–240	238–240 (ref. 94)
25	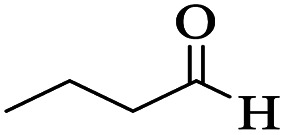	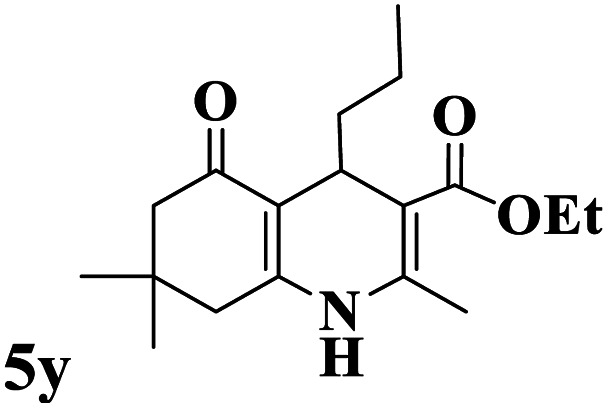	45	72	148–150	147–148 (ref. 57)

a Reaction conditions: dimedone or 1,3-cyclohexandione (1 mmol), ethyl acetoacetate or methyl acetoacetate (1 mmol), aldehydes (1 mmol), ammonium acetate (1.2 mmol) and catalyst (10 mg) at 90 °C.

b Isolated yields.

**Scheme 3:**
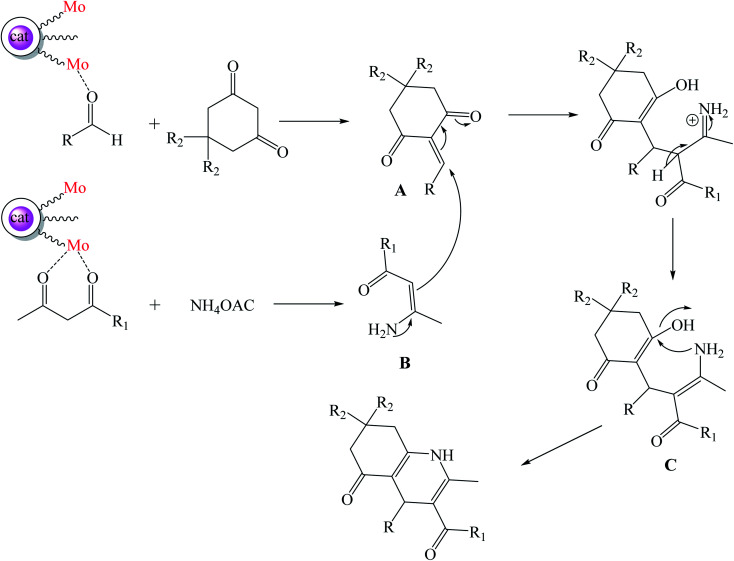
Proposed mechanism for the synthesis of polyhydroquinoline derivatives.

The plausible mechanism for the synthesis of polyhydroquinoline derivatives using Mo@GAA-Fe_3_O_4_ MNPs has been shown in Scheme 3. The metallic parts of the catalyst play a noticeable role in this organic transformation. Initially, Mo groups of the catalyst activate the C=O functional groups of aldehyde and facilitate the condensation with dimedone or 1,3-cyclohexandione and afford intermediate A. Then, Mo groups of the catalyst also activate C=O functional groups of ethyl acetoacetate or methyl acetoacetate and increase the activity of this group. The condensation of ethyl acetoacetate or methyl acetoacetate and ammonium acetate resulted in intermediate B. The Michael addition of intermediate A with intermediate B resulted in intermediate C. The achieved compound is unstable and is converted to the resulting product in subsequent dehydration and cyclization.

**Fig. 9:**
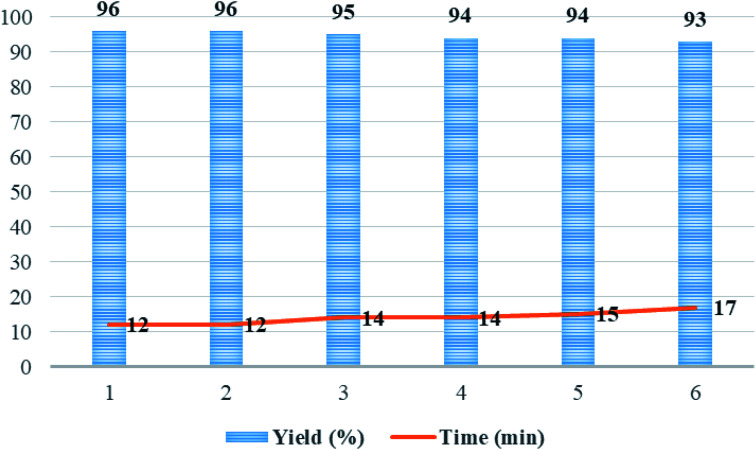
The recycling of Mo@GAA-Fe_3_O_4_ MNPs as catalysts under solvent-free conditions in the synthesis of 5p.

After completion of the reaction as indicated by thin layer chromatography (TLC), the catalyst precipitate was easily filtered off from the product using an external magnetic field, washed with water/ethanol (1 : 1) to eliminate the residual product, dried under the vacuum oven, and reused under the same experimental conditions. The recycled catalyst was reused with a negligible reduction in catalytic activity and the product yield for six runs (Fig. 9). The yields of the product 5p for each of the six runs were 96, 96, 95, 94, 94, and 93%, respectively.

To show the merits of the Mo@GAA-Fe_3_O_4_ MNPs in comparison with previously reported catalysts in the literature for the synthesis of polyhydroquinoline derivatives, some of the results are tabulated in Table 3. As indicated in Table 3, the low catalyst loading of Mo@GAA-Fe_3_O_4_ MNPs can develop a suitable methodology in terms of the reaction times and yields, green chemistry, and compatibility with the environment.

**Table 3:** Comparison of the results of the production of products 5n

Entry	Catalyst and conditions	Catalyst loading	Time (h)	Yield (%)	Ref.
1	Yb(OTf)_3_/EtOH/r.t.	5 mol%	5	90	57
2	(NH_4_)_6_[Mn^IV^Mo_9_O_32_]/EtOH/r.t.	2 mol%	1.5	90	95
3	MgMnO_3_/solvent-free/100 °C	8 mg	33 min	88	96
4	FePO_4_/EtOH/reflux	2 mol%	1	96	97
5	La_2_O_3_/TFE/r.t.	10 mol%	1	90	98
6	l-Proline/EtOH/reflux	10 mol%	5	92	99
7	Sc(OTf)_3_/EtOH/r.t.	25 mg	4	93	73
8	Cu(ii)-DCC-CMK-3/solvent-free/80 °C	30 mg	30 min	94	100
9	Al_2_(SO_4_)_3_/EtOH/reflux	10 mol%	4	92	101
10	Aluminized polyborate/solvent-free/100 °C	75 mg	20 min	92	102
11	Mo@GAA-Fe_3_O_4_ MNPs/solvent-free/90 °C	10 mg	13 min	95	This work

## 4. Conclusion

In summary, the Mo@GAA-Fe_3_O_4_ MNPs were synthesized as an effective, recyclable, and environmentally friendly heterogeneous magnetic nanocatalyst that catalyzed the synthesis of biologically and pharmacologically interesting functionalized polyhydroquinoline derivatives under solvent-free conditions. The catalyst can be magnetically recovered from the reaction media by an external magnet and reused several times without any significant changes in the reaction efficiency. Moreover, high yields of products, short reaction times, lower loading of the catalyst, clean procedure, easy work-up, and heterogeneous reaction conditions are several advantages of this straightforward and efficient strategy.

## Conflicts of interest

There are no conflicts to declare.

## Supplementary Material

## References

[cit1] Somorjai G. A., Park J. Y. (2008). Angew. Chem., Int. Ed..

[cit2] Liu Y., Zhang J., Zhang X., Li B., Wang X., Cao H., Wei D., Zhou Z., Cheetham A. K. (2016). J. Mater. Chem. A.

[cit3] Schmid G. , Nanoparticles: From Theory to Application, Wiley-VCH, Weinheim, 1st edn, 2004

[cit4] Duan S., Han G., Su Y., Zhang X., Liu Y., Wu X., Li B. (2016). Langmuir.

[cit5] Lu A. H., Salabas E. L., Schuth F. (2007). Angew. Chem., Int. Ed..

[cit6] Xing C., Liu Y., Su Y., Chen Y., Hao S., Wu X., Wang X., Cao H., Li B. (2016). ACS Appl. Mater. Interfaces.

[cit7] Niknam K., Saberi D. (2009). Tetrahedron Lett..

[cit8] Astruc D., Lu F., Aranzaes J. R. (2005). Angew. Chem., Int. Ed..

[cit9] Piao Y., Jang Y., Shokouhimehr M., Lee I. S., Hyeon T. (2007). Small.

[cit10] Kidwai M., Jain A., Bhardwaj S. (2012). Mol. Diversity.

[cit11] Shen X. F., Wang Q., Chen W. L., Pang Y. H. (2014). Appl. Surf. Sci..

[cit12] Li D. P., Zhang Y. R., Zhao X., Zhao B. X. (2013). Chem. Eng. J..

[cit13] Xu Y. Y., Zhou M., Geng H. J., Hao J. J., Ou Q. Q., Qi S. D., Chen H. L., Chen X. G. (2012). Appl. Surf. Sci..

[cit14] Zhang Y. R., Wang S. Q., Shen S. L., Zhao B. X. (2013). Chem. Eng. J..

[cit15] Zeng T., Chen W. W., Cirtiu C. M., Moores A., Song G., Li C. J. (2010). Green Chem..

[cit16] Arruebo M., Fernandez-Pacheco R., Ibarra M. R., Santamaria J. (2008). J. Pharm. Sci..

[cit17] Hong R. Y., Feng B., Liu G., Wang S., Li H. Z. (2009). J. Alloys Compd..

[cit18] Vayssières L., Chanéac C., Tronc E., Jolivet J. P. (1998). J. Colloid Interface Sci..

[cit19] Metz S., Thiel W. (2011). Coord. Chem. Rev..

[cit20] Hille R. (2013). Dalton Trans..

[cit21] Sanz R., Pedrosa M. R. (2009). Curr. Org. Synth..

[cit22] Jeyakumar K., Chand D. K. (2009). J. Chem. Sci..

[cit23] Sanz R., Pedrosa M. R. (2012). Adv. Org. Synth..

[cit24] Chand D. K. and Chakravarthy R. D., Molybdenum Chloride Oxide, in Encyclopedia of Reagents for Organic Synthesis, Wiley, 2012

[cit25] Sheikhshoaie I., Rezaeifard A., Monadi N., Kaafi S. (2009). Polyhedron.

[cit26] Bagherzadeh M., Haghdoost M. M., Ghanbarpour A. (2014). Inorg. Chim. Acta.

[cit27] Masteri-Farahani M., Tayyebi N. (2011). J. Mol. Catal. A: Chem..

[cit28] Masteri-Farahani M., Kashef Z. (2012). J. Magn. Magn. Mater..

[cit29] Divsalar N., Monadi N., Tajbakhsh M. (2016). J. Nanostruct..

[cit30] Sadri N., Moghadam M., Abbasi A. (2018). J. Mater. Sci.: Mater. Electron..

[cit31] Kakuchi R. (2014). Angew. Chem., Int. Ed..

[cit32] Domling A., Ugi I. (1993). Angew. Chem., Int. Ed..

[cit33] Kreye O., Toth T., Meier M. A. R. (2011). J. Am. Chem. Soc..

[cit34] Bae I., Han H., Chang S. (2005). J. Am. Chem. Soc..

[cit35] Zhu J. and Bienaymé H., Multicomponent Reactions, Wiley-VCH, Weinheim, 2005

[cit36] Moos W. H., Hurt C. R., Morales G. A. (2009). Mol. Diversity.

[cit37] Lamberth C., Jeanguenat A., Cederbaum F., De Mesmaeker A., Zeller M., Kempf H. J., Zeun R. (2008). Bioorg. Med. Chem..

[cit38] Magedov I. V., Kornienko A. (2012). Chem. Heterocycl. Compd..

[cit39] Akritopoulou-Zanze I. (2008). Curr. Opin. Chem. Biol..

[cit40] Dömling A., Wang W., Wang K. (2012). Chem. Rev..

[cit41] Touré B. B., Hall D. G. (2009). Chem. Rev..

[cit42] Trivedi A., Dodiya D., Dholariya B., Kataria V., Bhuva V., Shah V. (2011). Chem. Biol. Drug Des..

[cit43] Klusa V. (1995). Drugs Future.

[cit44] Murthy Y. L. N., Rajack A., Taraka-Ramji M., Jeson-Babu J., Praveen C., Aruna-Lakshmi K. (2012). Bioorg. Med. Chem. Lett..

[cit45] Sun C., Chen Y., Liu T., Wu Y., Fang T., Wang J., Xing J. (2012). Chin. J. Chem..

[cit46] Hilgeroth A. (2002). Mini-Rev. Med. Chem..

[cit47] Triggle D. J., Langs D. D., Janis R. A. (1989). Med. Res. Rev..

[cit48] Kawase M., Shah A., Gaveriya H., Motohashi N., Sakagami H., Varga A., Molnar J. (2002). Bioorg. Med. Chem..

[cit49] Mason R. P., Mak I. T., Trumbore M. W., Mason P. E. (1999). Am. J. Cardiol..

[cit50] Aruoma O., Smith C., Cecchini R., Evans P., Halliwell B. (1991). Biochem. Pharmacol..

[cit51] Chen Y. L., Fang K. C., Sheu J. Y., Hsu S. L., Tzeng C. C. (2000). J. Med. Chem..

[cit52] Roma G., Braccio M. D., Grossi G., Chia M. (2000). Eur. J. Med. Chem..

[cit53] Maguire M. P., Sheets K. R., McVety K., Spada A. P., Zilberstein A. (1994). J. Med. Chem..

[cit54] Ji S. J., Jiang Z. Q., Lu J., Loh T. P. (2004). Synlett.

[cit55] Ohberg L., Westman J. (2001). Synlett.

[cit56] Phillips A. P. J. (1949). J. Am. Chem. Soc..

[cit57] Wang L. M., Sheng J., Zhang L., Han J. W., Fan Z., Tian H., Qian C. T. (2005). Tetrahedron.

[cit58] Ko S., Yao C. F. (2006). Tetrahedron.

[cit59] Sabitha G., Reddy G. S. K., Reddy C. S., Yadav J. S. (2003). Tetrahedron Lett..

[cit60] Das B., Ravikanth B., Ramu R., Vittal Rao B. (2006). Chem. Pharm. Bull..

[cit61] Surasani R., Kalita D., Dhanunjaya Rao A. V., Yarbagi K., Chandrasekhar K. B. (2012). J. Fluorine Chem..

[cit62] Maheswara M., Siddaiah V., Damu G. L., Venkata Rao C. (2006). ARKIVOC.

[cit63] Heydari A., Khaksar S., Tajbakhsh M., Bijanzadeh H. R. (2009). J. Fluorine Chem..

[cit64] Song G., Wang B., Wu X., Kang Y., Yang L. (2005). Synth. Commun..

[cit65] Ko S., Sastry M. N. V., Lin C., Yao C. F. (2005). Tetrahedron Lett..

[cit66] Watanabe Y., Shiota K., Hoshiko T., Ozaki S. (1983). Synthesis.

[cit67] Ahankar H., Ramazani A., Joo S. W. (2016). Res. Chem. Intermed..

[cit68] Heravi M. M., Bakhtiri K., Javadi N. M., Bamoharram F. F., Saeedi M., Oskooi H. A. (2007). J. Mol. Catal. A: Chem..

[cit69] Kumar A., Maurya R. A. (2007). Tetrahedron.

[cit70] Kumar A., Maurya R. A. (2008). Synlett.

[cit71] Fardood S. T., Ramazani A., Moradi S. (2017). J. Sol-Gel Sci. Technol..

[cit72] Brietenbucher J. G., Figliozzi G. (2000). Tetrahedron Lett..

[cit73] Donelson J. L., Gibbs A., De S. K. (2006). J. Mol. Catal. A: Chem..

[cit74] Yarie M., Zolfigol M. A., Bayat Y., Asgari A., Alonso D. A., Khoshnood A. (2016). RSC Adv..

[cit75] Tamoradi T., Ghadermazi M., Ghorbani-Choghamarani A. (2018). Appl. Organomet. Chem..

[cit76] Evans C. G., Gestwicki J. E. (2009). Org. Lett..

[cit77] Ghorbani-Choghamarani A., Tahmasbi B., Moradi P., Havasi N. (2016). Appl. Organomet. Chem..

[cit78] Chen G. J., McDonald J. W., Newton W. E. (1976). Inorg. Chem..

[cit79] Abbaspour-Gilandeh E., Azimi S. C., Mohammadi-Barkchai A. (2014). RSC Adv..

[cit80] Abbaspour-Gilandeh E., Aghaei-Hashjin M., Yahyazadeh A., Salemi H. (2016). RSC Adv..

[cit81] Abbaspour-Gilandeh E., Yahyazadeh A., Aghaei-Hashjin M. (2018). RSC Adv..

[cit82] Khorshidi A., Tabatabaeian K., Azizi H., Aghaei-Hashjin M., Abbaspour-Gilandeh E. (2017). RSC Adv..

[cit83] Yahyazadeh A., Abbaspour-Gilandeh E., Aghaei-Hashjin M. (2018). Catal. Lett..

[cit84] Rad-Moghadam K., Azimi S. C., Abbaspour-Gilandeh E. (2013). Tetrahedron Lett..

[cit85] Maleki A., Kamalzare M., Aghaei M. (2015). J. Nanostruct. Chem..

[cit86] Peng H. N., Zheng D. G., Peng X. M. (2011). Asian J. Chem..

[cit87] Qin X. Y., Jin T. S., Zhou Z. X., Li T. S. H. (2010). Asian J. Chem..

[cit88] Moosavi-Zare A. R., Zolfigol M. A., Zarei M., Zare A., Afsar J. (2015). Appl. Catal., A.

[cit89] Nasr-Esfahani M., Elhamifar D., Amadeh T., Karimi B. (2015). RSC Adv..

[cit90] Zare A., Abi F., Moosavi-Zare A. R., Beyzavi M. H., Zolfigol M. A. (2013). J. Mol. Liq..

[cit91] Nasr-Esfahani M., Hoseini S. J., Montazerozohori M., Mehrabi R., Nasrabadi H. (2014). J. Mol. Catal. A: Chem..

[cit92] Sapkal S. B., Shelke K. F., Shingate B. B., Shingare M. (2009). Tetrahedron Lett..

[cit93] Reddy C. S., Raghu M. (2008). Chin. Chem. Lett..

[cit94] Maleki B., Tayebee R., Kermanian M., Ashrafi S. (2013). J. Mex. Chem. Soc..

[cit95] Amit R. S., Gavisiddappa S. G. (2009). Open Catal. J..

[cit96] Soleimani F., Salehi M., Gholizadeh A. (2020). Iran. J. Polym. Sci. Technol..

[cit97] Behbahani F. K., Homafar M. (2012). Synth. React. Inorg., Met.-Org., Nano-Met. Chem..

[cit98] Tekale S. U., Pagore V. P., Kauthale S. S., Pawar R. P. (2014). Chin. Chem. Lett..

[cit99] Karade N. N., Budhewar V. H., Shinde S. V., Jadhav W. N. (2007). Lett. Org. Chem..

[cit100] Ghafouri-Nejad R., Hajjami M. (2020). React. Kinet., Mech. Catal..

[cit101] Kulkarni P., Chil J. (2014). Chem. Soc..

[cit102] Aute D., Kshirsagar A., Uphade B., Gadhave A. (2020). Res. Chem. Intermed..

